# Evening Attendance at Dispensaries

**Published:** 1906-02-03

**Authors:** 


### EDITOR'S LETTER-BOX.

[Our Correspondents are reminded that prolixity is a great bar to publication, and that brevity of style and conciseness of statement greatly facilitate early insertion.]

### EVENING ATTENDANCE AT DISPENSARIES.

Mr. Harry Hems, a subscriber and visitor to the Exeter Medical Charities of many years' standing, writes as follows: The Exeter Dispensary was established in 1818, and at present treats from five to six thousand patients per annum. The inconvenience caused to some of the working classes by advice there only being available during the forenoons has suggested the advisability of opening the institution for these persons for an hour or two on two or three evenings each week.

Objecting to this, Mr. Charles E. Bell, of this city, one of our consulting surgeons, asserts no dispensary exists in this country where evening attention is available. Is it possible that the worthy gentleman in question is in error in this impression? If so, will someone be good enough to give the name of a dispensary or dispensaries where patients may receive the benefits of such charities after their ordinary working hours?

As a member of the Weekly Board of the Exeter Dispensary for thirty years, I crave and shall be grateful for information upon this point.

.·. At the Eastern, the Islington, the St. Pancras, and the Tower Hamlets Dispensaries an hour is set apart usually on two evenings in each week for the attendance of such patients as are unable to present themselves at other times. The whole question of evening attendance is fully dealt with in an annotation in the present week's issue, see page 299.—Ed. The Hospital.

